# Development of a Robust Read-Across Model for the Prediction of Biological Potency of Novel Peroxisome Proliferator-Activated Receptor Delta Agonists

**DOI:** 10.3390/ijms25105216

**Published:** 2024-05-10

**Authors:** Maria Antoniou, Konstantinos D. Papavasileiou, Georgia Melagraki, Francesco Dondero, Iseult Lynch, Antreas Afantitis

**Affiliations:** 1 Department of Chemoinformatics, NovaMechanics Ltd., Nicosia 1046, Cyprus; antoniou@novamechanics.com (M.A.); papavasileiou@novamechanics.com (K.D.P.); 2 Department of ChemoInformatics, NovaMechanics MIKE, 18545 Piraeus, Greece; 3 Entelos Institute, Larnaca 6059, Cyprus; francesco.dondero@uniupo.it (F.D.); i.lynch@bham.ac.uk (I.L.); 4 Division of Physical Sciences & Applications, Hellenic Military Academy, 16672 Vari, Greece; georgiamelagraki@gmail.com; 5 Department of Science and Technological Innovation, Università del Piemonte Orientale, 15121 Alessandria, Italy; 6 School of Geography, Earth and Environmental Sciences, University of Birmingham Edgbaston, Birmingham B15 2TT, UK

**Keywords:** PPARδ agonists, molecular docking, in silico modelling, machine learning, Isalos Analytics Platform

## Abstract

A robust predictive model was developed using 136 novel peroxisome proliferator-activated receptor delta (PPARδ) agonists, a distinct subtype of lipid-activated transcription factors of the nuclear receptor superfamily that regulate target genes by binding to characteristic sequences of DNA bases. The model employs various structural descriptors and docking calculations and provides predictions of the biological activity of PPARδ agonists, following the criteria of the Organization for Economic Co-operation and Development (OECD) for the development and validation of quantitative structure–activity relationship (QSAR) models. Specifically focused on small molecules, the model facilitates the identification of highly potent and selective PPARδ agonists and offers a read-across concept by providing the chemical neighbours of the compound under study. The model development process was conducted on Isalos Analytics Software (v. 0.1.17) which provides an intuitive environment for machine-learning applications. The final model was released as a user-friendly web tool and can be accessed through the Enalos Cloud platform’s graphical user interface (GUI).

## 1. Introduction

PPARs are members of the nuclear receptor (NR) superfamily of proteins, whose functions are essential for cell signalling, survival, and proliferation, which comprises 48 members in humans [1] and function as ligand-activated transcription factors. Their role is central in the regulation of diverse biological processes, encompassing immune system function, development, reproduction, and homeostasis [2], involving the control of gene expression related to fatty acid utilisation and storage [3]. Target gene regulation is achieved by PPAR binding to characteristic sequences of DNA bases, called peroxisome proliferator response elements (PPREs). PPREs are active as heterodimers with the receptor for 9-cis-retinoic acid (retinoid X receptor or RXR), and thus play a critical role in modulating the actions of hormones and ligands. Furthermore, PPARs participate in various cellular processes, including glucose utilisation, cell proliferation, cell differentiation, inflammatory responses, and adipogenesis [4]. Depending on their tissue expression, they are classified into three subtypes, namely PPARα, PPARγ, and PPARβ/δ [5], which also reflects their distinct physiological roles.

Although the PPAR subtypes exhibit a significant degree of amino acid sequence similarity, they vary in ligand selectivity and target genes in a species-specific manner [6]. For example, PPARδ exhibits significant expression levels in organs characterised by elevated rates of oxidative metabolism, such as the heart, skeletal muscle, and liver, while playing a regulatory role in the utilisation of fatty acids and glucose, as well as in antioxidant defence mechanisms [7].

Several attempts have been previously reported in the literature to construct QSARs for the establishment of statistically significant correlations for the prediction of PPAR agonists’ behaviour [8]. Specifically, classical (1D) and (2D)-QSAR models [9,10,11] were developed using a dataset evaluated by Wickens et al. [12], linking molecular properties and structural characteristics, respectively, to the activity of the compounds, with the predictions being confirmed through docking methods. In addition, QSAR modelling was represented using the three-dimensional (3D) properties of the ligands to predict the biological potency of PPARδ receptors by exploiting methods such as comparative molecular field analysis (CoMFA), which provides a visual display of the active centres in compounds that indicates the fragments contributing maximally to the activity profile of the compounds, and comparative molecular similarity indices analysis (CoMSIA), which expresses the fields in terms of molecular similarity indices rather than the usually applied Lennard–Jones- and Coulomb-type potentials, as used in CoMFA [13,14]. Other studies [15,16] employed a different dataset [17] comprising 34 PPARδ partial agonists, for the establishment of hologram QSARs (HQSARs) by using molecular holograms as variables for their predictive schemes. Lastly, Daadoosh et al. [18] employed a machine-learning method, iterative stochastic elimination (ISE), to perform the virtual screening of over 1.5 million compounds and identified thirteen highly selective PPARδ agonists [19]. It was apparent from these studies that the inclusion of molecular docking calculations in the models appears to ameliorate their poor predictive performance in the absence of the molecular docking information.

In the present study, we introduce a robust predictive model utilising a set of 136 novel PPARδ agonists that, according to the new OECD definition, includes per- and polyfluoroalkyl substances (PFAS) [20]. The model integrates diverse structural descriptors with docking calculations to predict the biological activity of PPARδ agonists, adhering to the criteria outlined by the OECD for the development of QSAR and read-across models. Concentrating specifically on small molecules, the model aids in identifying highly potent and selective PPARδ compounds, employing a read-across concept to delineate the chemical neighbours of the compound under investigation and thus to classify it as active or non-active based on their biological potency score.

## 2. Results

**Figure 1:**
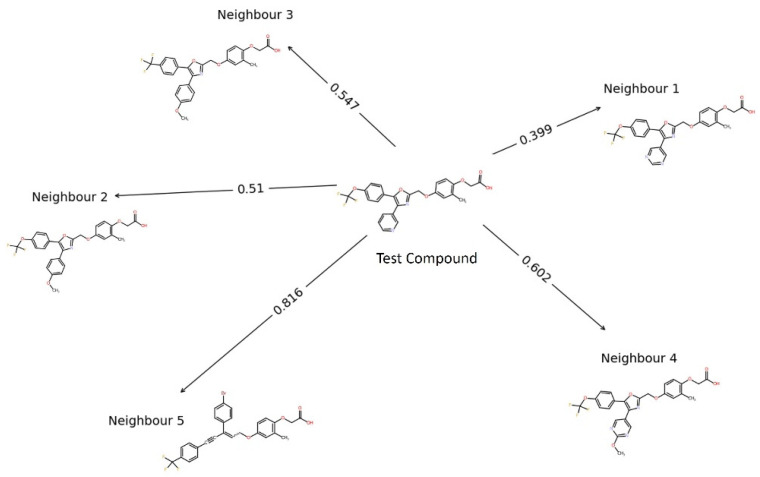
Network of a test compound (PubChem CID: 44627413) with Euclidean distances from its five closest neighbours.

The techniques mentioned in the Section 4 for the development of the predictive model were implemented in the Isalos Analytics Platform. First, the initial dataset of 136 novel compounds was derived from the PubChem public repository using the Enalos+ KNIME node ‘Main PubChem’. Each small molecule or compound was accompanied by an extensive set of 777 molecular descriptors that encode their structural, topological, and geometrical characteristics, along with a calculation of their binding affinity for the human PPARδ protein structure. The dimensionality of the data was reduced after numerous descriptors were excluded from the set using the ‘Remove Column’ function and a low variance filter (20%). The 245 remaining descriptors’ values were transformed with a Gaussian normalisation function into a new set of values that lie on a similar scale and whose mean is zero and standard deviation is one. A clustering technique was employed for the distinction of the novel molecules into two classes that represent biological potency. Through a greedy algorithm, the number of input descriptors was further reduced and the most relevant descriptors that exhibit optimal correlation to the target variable were distinguished. Following the pre-processing steps, a kNN classification algorithm was used as the modelling methodology, since it allows the observation of the five neighbouring instances of each test compound from the training set. This read-across approach allows the exploration of the adjacent chemical space of the compound under study [21], wherein the closest five neighbours are more likely to share physicochemical properties and structural patterns with the molecule of interest or test compound (Figure 1).

### 2.1. Interpretation of the Selected Descriptors

As mentioned above, the variables that were most pertinent to the modelling target were selected from a pool of 777 molecular descriptors after the ‘BestFirst’ function in Isalos was applied to the training dataset. Since the descriptors are mathematical representations of the molecules [22], the interpretation of the selected variables grants insight into the most significant factors that control the behaviour of chemicals against the PPARδ nuclear receptor. The eleven favoured descriptors, presented in Table S1, encode information mainly on the compounds’ bulk characteristics, their autocorrelation, and topological indices.

Firstly, the Broto–Moreau spatial autocorrelation descriptor (ATSe,7), which emerged as the most significant descriptor overall, is a measure whereby the atoms of a molecule are represented by an atomic property such as the Sanderson electronegativity [23,24]. It provides information on how the atomic property is distributed on the topological structure of the molecule, thus a higher electronegativity distribution within the molecule contributes to the biological activity of PPARδ. The total information content (TIC_m_) was also selected, which quantifies the complexity of a knowledge graph. Higher values amount to higher molecular graph complexity and highest effect concentrations (EC_50_s), evidenced from the positive correlation coefficient between the descriptor and the associated biological activity of the test compound.

The Burden eigenvalues are also among the highly influential descriptors. This descriptor is computed as a solution to the characteristic equation of the Burden connectivity matrix (B), whose elements correspond to a topological distance between pairs of atoms [25], and its diagonal elements (Bii) are given by the van der Waals volume values. Another important descriptor correlated with PPARδ activity is the sum of topological distances between the vertices of oxygen atoms and fluorine or sulphur atoms, calculated as the row sum of the distance matrix. Topological distances are the number of edges along the shortest path between two specific atoms, measuring the number of involved bonds [26].

Last but not least, the Kier shape index (S2k) was proven as a valuable variable that describes the shape of the molecule in terms of counts of two bond paths [27]. It captures the degree of star graph-likeness and provides information about the branching and the flexibility of the molecular structure. Higher S2k values indicate a greater degree of flexibility within the molecules. According to Xu et al. [28], PPAR activation is effective when the linked compound is flexible, thus less pliable compounds exhibit reduced potency (and thus have higher EC_50_ values). Even though we highlight the features that describe the biological system in an effective manner, further validation against experimental data is needed to establish meaningful correlations between the above-mentioned descriptors and the biological potency.

### 2.2. Model Validation

#### 2.2.1. Metrics and Statistics

Assessing the predictive performance of the model using several statistical criteria ensures that it can classify the instances effectively. After the implementation of the classification machine-learning algorithm, different statistics were employed for the evaluation of the model, based on the number of correct predictions and misclassifications of the test set [29]. Provided that the model aims to predict the potency class of a target compound, characterising it as either “active” or “inactive”, the problem boils down to a binary classification one.

Therefore, a confusion matrix for the test set is presented, which is essentially a table recording the number of true positive (TP), true negative (TN), false positive (FP) and false negative (FN) predictions in comparison with the actual classes of the agonists (Table 1).

Based on the confusion matrix, various classification performance indications can be obtained, including accuracy, sensitivity, and precision, all synopsized in Table 2. The measurements for the goodness-of-fit and predictivity were higher than 0.80 when applying the kNN algorithm, with an optimised value of k = 5 to the test set, which denotes the ability of the model to accurately capture patterns and return reliable predictions.

#### 2.2.2. Internal and External Validation

**Figure 2:**
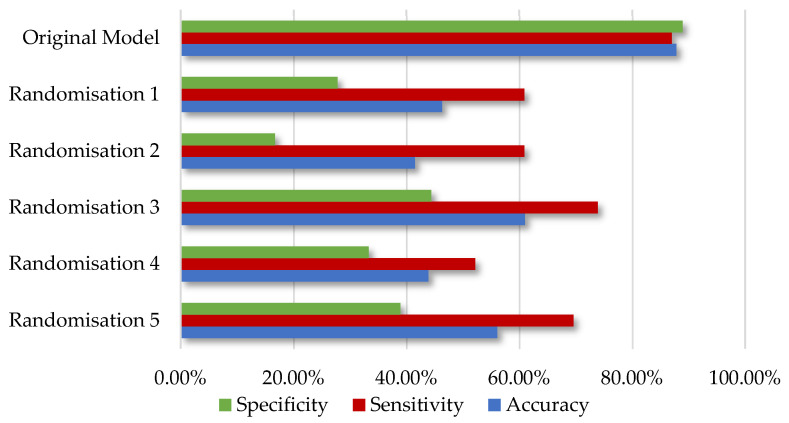
The predictive power of the original model compared with the models obtained from the five Y-randomization tests.

Internal validation was performed through the Y-randomisation procedure in order to ensure the robustness of the predictive model [30]. Specifically, the observed target feature’s values are randomly assigned to other compounds; thus, the original values of the descriptors now correspond to a different endpoint variable. Provided that the original model is robust, when it is applied on the test set it is expected that the predicted values are not close to the confounding ones, which is verified through the inadequate performance of the model. This technique was performed using the ‘Y-randomization’ node in KNIME contained in Enalos+ [31]. Calculations were repeated for five randomisations, ensuring that the model was not based on chance correlation and overfitting. When the algorithm was trained on disarranged targets, the predictive performance of the obtained models was statistically low, whereas the validation measurements of the original model were adequate (Figure 2). Specifically, the accuracy values derived fluctuated between 41.5% and 61.0% and were significantly lower compared to the accuracy value of 88.9% of the original model.

For external validation, the original subset was partitioned into the training set, which was used during model development, and the test set, which was used solely for validation. More precisely, the developed model was applied to the test set, which was not included in the development process and was later involved during the model’s performance assessment. This technique validates that the read-across model’s performance is satisfactory on unseen data that were not involved in the construction of the classifier.

### 2.3. Applicability Domain

The domain of applicability (APD) is defined after model validation and determines the area of reliable or unreliable predictions. It is essential for describing the limitations of a model and the degree of similarity between the compound of interest and the model training set, as determined by different approaches. A distance-based method is used in this work, which involves similarity measurements based on the Euclidean distances among all training data, compared to a predefined APD threshold [32,33].

At first, the average value of all Euclidean distances is calculated and then the set whose distances are lower than the average value are excluded from further calculations. Next, a new average value (d) and the standard deviation (σ) of the remaining distances sets is determined, thus the APD threshold is calculated as:

APD = zσ + d, (1)

where, z is an empirical parameter whose default value is 0.5. In the case that the distance from an external compound to its nearest neighbour (among the test set data) is smaller than the APD threshold, then the prediction is considered reliable. The APD thresholding was performed in the Isalos platform, Statistics → Domain—APD. The selected APD model, developed from the training subset, was employed from Analytics → Existing Model Utilisation in order to be applied to the test subset. The obtained APD threshold value was equal to 3.682, while the predictions were regarded as reliable for all compounds included in the test set. Table S2 of the Supplementary Information File includes the selected descriptors, an indication of the actual class of each compound in the testing set, and the prediction obtained from the model.

### 2.4. Model Availability

In order to accelerate the assessment of small molecules and their activity towards the PPARδ nuclear receptor, the read-across predictive model was disseminated as a publicly available web application in the Enalos Cloud Platform. Several fully validated cheminformatics models [34,35] are hosted by the Enalos Cloud Platform, supporting the scientific community by making the predictive workflows easily accessible to anyone interested. The model’s functionality can be easily accessed through a user-friendly interface that requires limited input, and no coding skills, in order to provide predictions.

**Figure 3:**
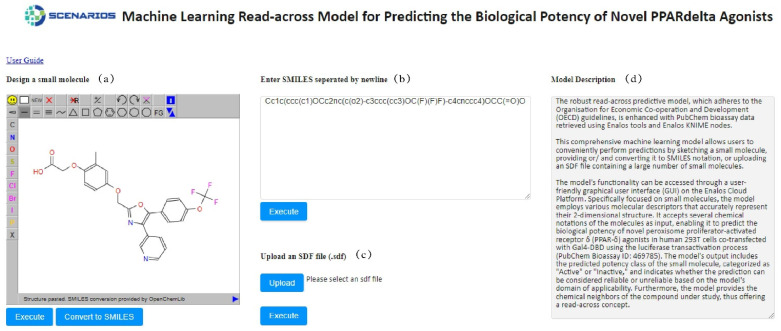
PPARδ environment in the Enalos Cloud Platform: The Design Molecule field for input compounds (**a**), the SMILES (**b**) and the SDF (**c**) field for input compounds, along with a brief description of the model (**d**).

Figure 3 portrays the initial interface, where a brief description of the model development is given, along with three different ways to insert compounds and initiate predictions. Either the SMILES notations can be entered manually or an SDF file that contains the structure of one or multiple compounds can be browsed and uploaded by the user. As a further option, users can use a drawing interface (Figure 3d) to design the molecule of interest. In the sketcher field, the user can also transform the initial molecule by adding different functional groups such as alkanes, amines and amides, benzene rings etc., or more complex chemical structures such as steroids and amino acids.

**Figure 4:**
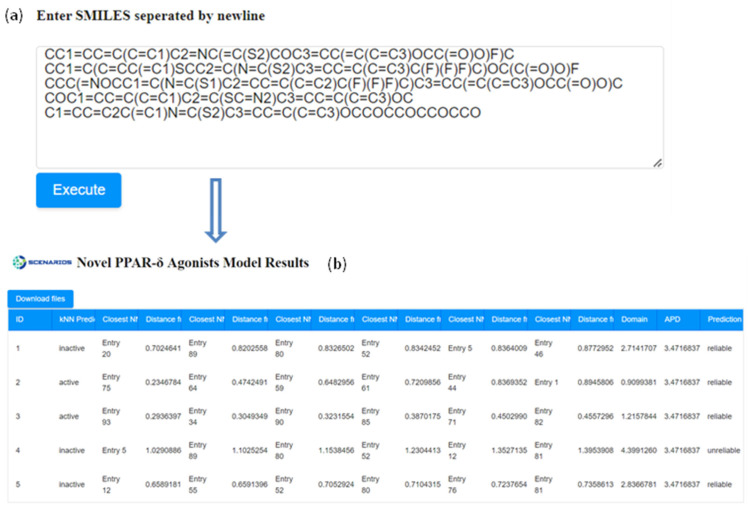
Entering the SMILES notations of five different compounds as input to the web application (**a**) and the generated output page (**b**). Out of the five compounds tested, the kNN algorithm identified only the two PFAS congeners (CID 54764927 and 51346913) as active.

For the demonstration of the tool’s functionality, five compounds of interest—including two substances with a perfluorinated methyl group (−CF_3_), i.e., PFAS—were selected from the PubChem library (CIDs: 155547595, 54764927, 51346913, 46230234, 137464756). The selected chemicals share at least 95% Tanimoto similarity with active compounds from the initial dataset, and their SMILES notations (Figure 4a) were extracted with the Enalos+ ‘Main PubChem’ node in KNIME. A prediction is generated within seconds, and the output includes a table that presents the classification of the compound’s activity, the five nearest neighbours of the input compound from the training set, and the Euclidean distances from each of the neighbours (Figure 4). The distance of each submitted compound calculated according to the APD of the model is presented, along with an indication of the reliability of each prediction. As presented in Figure 4b, when the calculated domain of the small molecule is higher than the APD threshold, the web application highlights that the prediction is not reliable. As seen from this case study, the read-across model can be used within a virtual screening framework to identify whether similar chemicals can be potentially used as activators of the PPARδ receptor.

**Figure 5:**
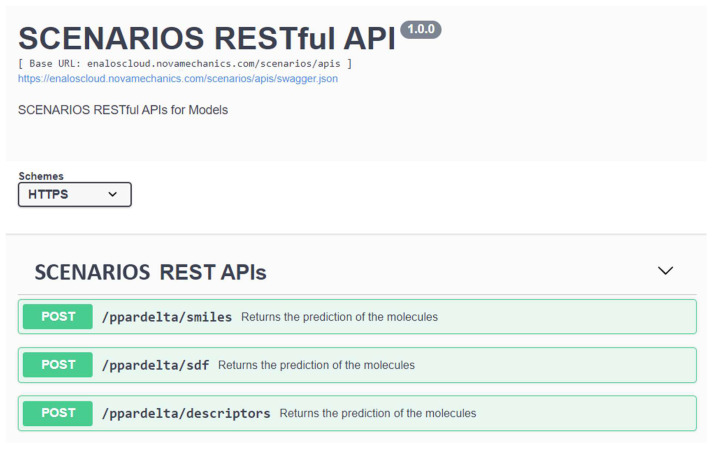
The REST API environment (accessed on 11 January 2024) for the PPARδ agonist bioactivity prediction.

In order to enhance the accessibility and programmability of the predictive model, a Representational State Transfer (REST) application programming interface (API) was incorporated (https://enaloscloud.novamechanics.com/scenarios/swagger-ui/index.html, accessed on 11 January 2024). This method is useful as it allows seamless integration of the computational workflow into various systems and platforms and enables users to explore the capabilities of the model without direct access to the original workflow. Users are, therefore, able to incorporate the model into their own workflows through the API (Figure 5). It is further used to communicate with the Isalos Analytics Platform to exchange data for the straightforward execution of the model. The API was implemented using the POST request method, since it is suitable for transferring substantial amounts of structured input data securely. The submission of a tuple of data input (i.e., containing either a single or multiple SMILES string(s) of the desired compound(s)) in JSON format is needed to use the PPARδ agonists bioactivity API:

  [

  {

  “smiles”: “Cc1c(ccc(c1)OCc2nc(c(o2)-c3ccc(cc3)OC(F)(F)F)-c4cnccc4)OCC(=O)O”

  }

]
The user is able to make a request through a data transfer software such as Client URL:
 
curl -X POST “https://enaloscloud.novamechanics.com/scenarios/apis/ppardelta/smiles” -H “accept: application/json” -H “Content-Type: application/json” -d “[ { \”smiles\”: \”Cc1c(ccc(c1)OCc2nc(c(o2)-c3ccc(cc3)OC(F)(F)F)-c4cnccc4)OCC(=O)O\” }]”
 
and obtain the corresponding results of the GUI environment, as seen in Figure 4. The returned response includes class prediction, the closest neighbours, and the Euclidean distances from the molecule in question, and the APD indicating the reliability of the prediction:
 
[
{
“id”: “cluster_0”,
“idNN1”: “Entry 20”,
“distNN1”: 0.7024641202204505,
“idNN2”: “Entry 89”,
“distNN2”: 0.8202558963827292,
“idNN3”: “Entry 80”,
“distNN3”: 0.8326502973790398,
“idNN4”: “Entry 52”,
“distNN4”: 0.8342452655891001,
“idNN5”: “Entry 5”,
“distNN5”: 0.8364009145055961,
“idNN6”: “Entry 46”,
“distNN6”: 0.8772952879358424,
“domain”: 2.714170703054797,
“apd”: 3.4716837408236625,
“predictionReliablility”: “reliable”,
“knnprediction”: “inactive”
}
]

## 3. Discussion

In summary, in the present study an in silico predictive model that correlates novel PPARδ agonists’ two-dimensional chemical structures to their biological potencies in terms of nuclear receptor activation (PPARδ) was successfully developed. PPARδ, a subtype of the nuclear receptor superfamily, plays a pivotal role in regulating cellular metabolic functions and in modulating diseases associated with changes in lipid and glucose homeostasis. The search for highly potent and selective compounds that act as PPARδ activators is still ongoing [36]; hence, the development of computational methods that assist in the identification of such compounds is crucial.

The predictive model in this study uses an initial dataset sourced from the PubChem BioAssay public repository that consists of 136 novel molecules tested in human 293T cells co-transfected with Gal4-DBD via a process called luciferase transactivation. The chemical structure of each compound of the dataset is represented through a comprehensive set of 777 molecular descriptors, generated using the EnalosMold2 specialised module in KNIME. Apart from the structural properties of the molecules, docking calculations were included as a supplementary variable. All analysis steps, including the normalisation of the descriptors, the selection of the most correlated variables, the algorithm selection, and the validation of the final model were executed within the Isalos Analytics software (v. 0.1.17), an advanced platform for machine-learning applications. The model underwent internal and external validation, through the use of different subsets for training and testing and Y-randomization tests, demonstrating strong performance. Fully adhering to the guidelines posed by the OECD, the domain of applicability was described, defining the region in the chemical space where the generated predictions can be trusted. The interpretation of the molecular descriptors’ influence on the compounds’ biological activity is discussed. While the descriptors’ effect on the biological potency was emphasised, full comprehension of their effects on the biological potency requires additional experimental validation. The model is fully documented via a QMRF (Table S3) report, which was prepared for the reporting of the key information on this read-across model for regulatory use.

Although successful attempts to derive statistically significant relationships on the biological activity of PPARδ have been reported in the past, the present work applies a different modelling approach. In comparison to the use of traditional QSAR methodologies, this work enables the categorising of an unknown compound into ‘active’ or ‘inactive’ and introduces a read-across paradigm that provides information on the five closest instances (neighbours) from the training data. The separation of the compounds into two distinct classes facilitates rapid decision-making in early drug discovery. While the current study is tailored to distinguishing compounds for the activation of PPARδ, the read-across methodology can be adapted for the identification of small molecules that can act as agonists or antagonists against other biological targets. The proposed read-across methodology can be extended across other proteins or enzymes to describe structure–activity relationships with potential regulators. A similar in silico approach can be implemented for other nuclear receptors, such as the PPARα or PPARγ ligand-activated transcription factors, starting from the identification of experimental datasets that identify the agonists and antagonists of the target nuclear receptors. Recently, a computational tool [37] was developed for the prediction of chemical molecules’ binding class to multiple nuclear receptors, but the previous tool does not employ the read-across framework and does not distinguish between agonist and antagonist compounds regarding the PPARδ receptor.

Additionally, the present work enables export of the read-across model as a web tool via the Enalos Cloud Platform. The web tool can be easily accessed through the following link: http://www.enaloscloud.novamechanics.com/scenarios/ppardelta/ (accessed on 11 January 2024). This comprehensive model allows users to provide input data by sketching a small molecule, entering and converting it to SMILES notation, or by uploading an SDF file containing a large number of small molecules. The web application offers the possibility to use the predictive capabilities of the model from anywhere, assisting scientists and researchers in the continuing process of detecting PPARδ activators.

## 4. Materials and Methods

**Figure 6:**
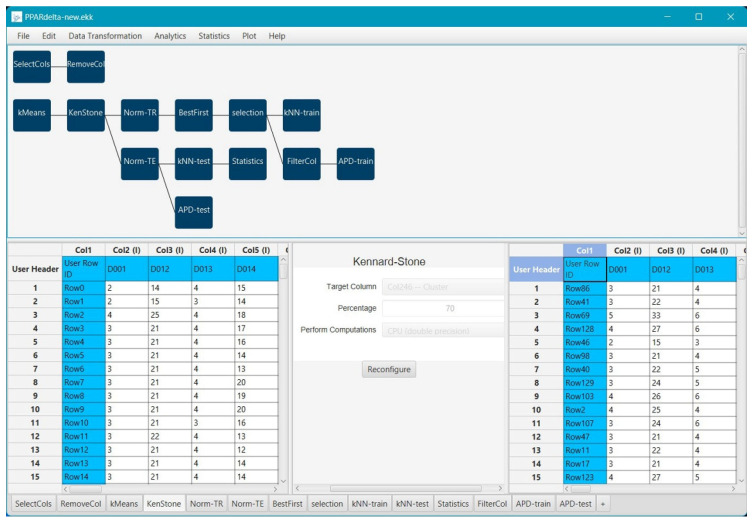
Implementation of the model development process in Isalos Analytics Platform.

The comprehensive analysis for the development of the read-across predictive model was performed with the Isalos Analytics Platform [38]. Isalos is a simple, straightforward software developed by NovaMechanics Ltd (Nicosia, Cyprus) (https://isalos.novamechanics.com/, accessed on 4 December 2024), which allows the implementation of machine-learning workflows without requiring coding skills. The Isalos Platform provides a practical interface through the use of menus, tabs, and buttons, while each tab acts as a node and allows the transformation and transition of data in tabular form. All analysis steps, including data preparation, feature selection, algorithm building, and model validation, were performed using the special functions encoded in the software. Leveraging the built-in functions, along with the Enalos+ proprietary nodes [31] accessible through the KNIME Analytics Platform, results in a combined workflow, as illustrated in Figure 6. The final predictive model is fully validated according to the OECD guidelines [39] and its key information was summarised and reported using the QSAR Model Reporting Format (QMRF) template, following the guidance of the Joint Research Centre and the European Centre for Validation of Alternative Methods [40]. The completed reporting template can be found in the electronic Supplementary Information File (ESI Table S3).

### 4.1. Dataset

Epple et al. [41] performed a high throughput screening (HTS) of approximately 1 million chemical compounds, defining hits as molecules that induced luciferase activity and utilising this assessment as an indicator of agonist activity against the human PPARδ ligand binding domain. The luciferase gene encodes a 61-kDa enzyme that oxidises D-luciferin in the presence of ATP, oxygen, and Mg^2+^, yielding a fluorescent product that can be quantified by measuring the released light via a luminescence assay. The molecules were tested in a human embryonic kidney cell line, 293T, co-transfected with a chimeric plasmid with the yeast GAL 4 DNA-binding domain (DBD). The dataset was retrieved from PubChem BioAssay, a public repository for the biological activities of small molecules and small interfering RNAs hosted by the National Institutes of Health (NIH), under the numeric identifier AID 469785 [42]. All 136 retrieved oxazole-based compounds from the initial dataset are accompanied by a standardised measure of potency, the half maximal effective concentration (EC_50_), which determines the agonist concentration needed to elicit half of its maximum biological effect, in this case cytotoxicity to human embryonic kidney cells. The EC_50_ value is inversely related to a compound’s potency [43]. It is important to note that Garcia et al. [13] and Nandy et al. [44] utilised the same experimental dataset to apply 2D and 3D QSAR methodologies for the assessment of the biological activity of PPARδ agonists. However, they used a subset that comprised just above 100 compounds, in contrast to the entirety of the dataset as used in this work. The inclusion of the complete dataset ensures the generalizability of our model and provides a broader applicability domain. Additionally, while the other studies focus on the derivation of regressive QSARs, aiming to predict the value of a potency metric, the read-across model developed in this study deploys a different modelling approach, classifying the small compounds into the ‘Active’ and ‘Inactive’ categories and enabling prediction of which class an unknown small molecule fits into, based on the applicability domain.

### 4.2. Calculation of Descriptors

The Mold2 [45] software package (version 2.0), accessed through the Enalos+ node ‘EnalosMold2′ [31] node available in KNIME, was used for the retrieval of molecular descriptors representing characteristics of the small molecules. Requiring only the ‘Simplified Molecular Input Line Entry System’ (SMILES) notations as input, in the Structure Data File (SDF) format, Mold2 calculates 777 molecular descriptors based on the one-dimensional (1D) and two-dimensional (2D) structure of each compound. The calculated 1D descriptors are related to counts of atoms, and the 2D descriptors mainly refer to bonds and functional groups, physicochemical properties, autocorrelation, charge, connectivity, and topological features of the molecules.

### 4.3. Data Pre-Processing

Data modification is a crucial step in data analysis, as it allows the cleaning, reduction, and transformation of data as a means of eliminating noisy data and improving the performance of machine-learning algorithms. Firstly, duplicates containing more than 80% of the same repeated values of particular parameters were removed from the dataset using the Isalos’ ‘Remove Column’ function. Furthermore, the extracted raw data were pre-processed with a low variance filter in order to reduce the dimensionality of the dataset and filter out descriptors that have least impact on the target variable. An upper bound of 20% was chosen; thus, descriptors whose variance fell below the threshold were excluded from the following steps.

### 4.4. Clustering into Distinct Classes

Since the initial dataset’s potency score, EC50, was available as a continuous variable without predefined classes, an unsupervised clustering method was used to explore the natural groupings of the unlabelled compounds. The k-means algorithm was chosen to perform an initial analysis and divide the instances into appropriate groups according to their activity indicators. The k-means is a useful method for partitioning variables into k separated clusters, where each cluster is represented by its centroid average [46]. The algorithm begins by randomly selecting k observation compounds as the initial centroids, then proceeds by assigning each observation to the cluster whose centroid is closest to it. Euclidean distance is used as a distance-calculating method. The centroids are then recomputed as the average of the observations allocated to the cluster, and this process is repeated until the assignment of observations to clusters no longer changes [47].

By employing a clustering method, an initial partitioning is created, assuming that k-means partitions (*k* = 2) now distinguish all data as belonging to one of two biological activity classes (active or inactive) based on their log-transformed EC_50_ values given in micromolar (μΜ) units. The logarithmic transformation of the values was preferred, in order to reduce the skewness of the data [48]. Each cluster represents an activity class; therefore, the original regression problem is reduced to a simpler classification problem, assigning a class label to each observation. The higher the EC_50_ value, the more the concentration of a compound is required to obtain a 50% effect inducement, and the lower the potency. This designates that the compounds assigned in the cluster with a centroid of log(EC50) = 0.224 are considered inactive and those included in the cluster with a centroid of log(EC50) = −1.674 are regarded as active. The coverage of the two clusters is 58 and 78 compounds, respectively, portraying a broadly balanced dataset.

Feature scaling is another fundamental pre-processing step, which is performed after the k-mean clustering and normalises the range of the independent attributes. The selected method for normalisation is z-score scaling, used to transform the data to have a mean of zero and a unit standard deviation (Gaussian-distributed). In the Isalos Analytics Platform, Gaussian standardisation is available in Data Transformation → Data Manipulation → Z-score. Prior to further modelling, the collected data were also divided into two subsets, the training and test datasets, as an external validation procedure. The two representative sets were split 70/30%, respectively, using the Kennard–Stone algorithm [49,50], available in Isalos.

### 4.5. Variable Selection

The set of descriptors produced by the Mold2 Enalos+ is characterised by its considerable size and diversity, indicating the presence of numerous descriptors that may be redundant or unrelated to the forthcoming analysis. This issue renders feature selection a necessary step prior to modelling. By using ‘Best First’ as a search method, the most important variables out of the Mold2-derived descriptors are selected (from the 777 available descriptors) based on the training set to be included in the model. This method uses a greedy algorithm, starting with an empty feature set and iteratively adding or removing features based on certain criteria, in order to choose the successor out of all combinations, and it is implemented using the Isalos Analytics Platform through Analytics → Feature selection. Table S1 in the Electronic Supplementary Information (ESI) describes the 11 selected attributes as per the Handbook of Molecular Descriptors [22]. As an additional descriptor, the molecular docking scores of the small molecules to the PPARδ receptor were also taken into consideration. Including the binding affinity values in the dataset seems to improve the results and contributes to the variability of the data, since it increases the dataset and provides a more thorough examination of the interactions with the receptor.

### 4.6. Molecular Docking Calculations

**Figure 7:**
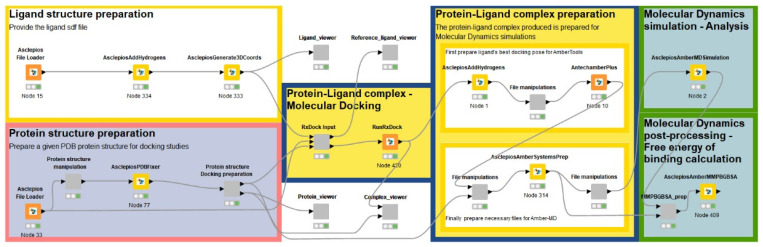
The Enalos Asclepios KNIME pipeline for automation of the drug discovery pipeline, applied here to screening PPAR biological activity.

Molecular docking calculations were conducted using the Vina-GPU 2.0 software [51,52], on the set of compounds retrieved from PUBCHEM, by employing the PPARδ homo sapiens structure (PDB ID: 3TKM [53]). The structural preparation of PPARδ involved the Enalos Asclepios KNIME pipeline [54] (Figure 7), encompassing tasks such as the addition of missing residues, removal of heteroatoms, replacement of non-standard residues, and addition of heavy atoms using the PDBFixer software (version 1.9) [55]. Hydrogen atoms were subsequently added with the pdb4amber utility of AmberTools21 [56]. Ligand preparation was performed using the Enalos Asclepios KNIME pipeline, incorporating steps such as the addition of missing hydrogen atoms via Open Babel [57], setting the pH value at 7.4, and conversion of 2D structures to 3D through energy minimization using AsclepiosGenerate3DCooords [54]. The Enalos Asclepios KNIME nodes and workflow used in this study are proprietary to NovaMechanics Ltd and require a licensing agreement for access.

The PPARδ structure and 136 compounds for docking were prepared using AutoDock Tools 1.5.7 python libraries [58,59], with partial atomic charges assigned based on the Kollman United Atom and Gasteiger–Marsili schemes, respectively, by previously merging non-polar hydrogens to heavy atoms. For the ligands, the torsion tree and the rotatable/non-rotatable bonds present were also set [58,59]. Calculations involved a docking box with dimensions set at 25 Å × 25 Å × 25 Å and 1 Å spacing, placing the centre of the grid at the centre of mass coordinates of the crystallographic ligand, which was used as a reference. The number of threads was set equal to 8000.

### 4.7. Model Development

Among the methodologies tested for the establishment of a correlation between the structural properties of, and the biological response to, PPARs, the k-Nearest Neighbours (kNN) classification algorithm emerged as the most appropriate. It is an easily implemented supervised machine-learning technique that is utilised in resolving problems for both continuous and categorical endpoints. The kNN algorithm operates on the principle of identifying the k-number of training data points that are most proximate to a new, unclassified observation based on Euclidean distances, and subsequently assigning the class label that is most frequently represented among the k-nearest neighbours [60].

kNN is considered a ‘lazy’ learning algorithm since it simply uses the training data for classification instead of building a new model beforehand for new data points and can be used under a read-across framework [61,62]. The optimised value of k, which denotes the number of nearest neighbours to consider, was set at k = 5 and the inverted distance was used as the weighting factor for the nearest k points. An overview of the flowchart conducted in Isalos is presented in Figure 6, where all the significant steps of the analysis were implemented with the software’s specified functions.

## 5. Conclusions

In this study, a dataset of novel thiazole- and oxazole-based compounds was enhanced with binding affinity calculations for the development of a read-across QSAR model to predict their activation of nuclear receptor PPAR. The predictive model attempts to predict the biological activity of small molecules and helps to identify highly potent and selective compounds that act as PPARδ agonists. Using a combination of molecular descriptors that correspond to the different physicochemical, topological, and structural characteristics of a compound, and defining the domain of applicability for new predictions, this model was carefully developed, validated, and documented to adhere to OECD guidelines for QSARs, including provision of a QMRF report. The read-across model is readily available as a public web application within the Enalos Cloud Platform, a valuable resource for predictive workflows for the assessment of small molecules. A REST API environment is also provided in order to complement the model and offer the users a means to augment its potential.

## Figures and Tables

**Table 1:** Confusion Matrix summarising the number of correct and incorrect predictions from the test set.

Class	Predicted Active	Predicted Inactive
Actual Active	20	3
Actual Inactive	2	16

**Table 2:** Accuracy statistics of the predictive model.

Metric	Metric Formula	Metric Value
Accuracy	$\frac{\mathrm{TP}+\mathrm{TN}}{\mathrm{TP}+\mathrm{TN}+\mathrm{FP}+\mathrm{FN}}$	87.8%
Sensitivity	$\frac{\mathrm{TP}}{\mathrm{TP}+\mathrm{FN}}$	87.0%
Precision	$\frac{\mathrm{TP}}{\mathrm{TP}+\mathrm{FP}}$	90.9%
F1-Score	$\frac{2\mathrm{TP}}{2\mathrm{TP}+\mathrm{FP}+\mathrm{FN}}$	88.9%
Matthews Correlation Coefficient	$\frac{\mathrm{TP}\times \mathrm{TN}-\mathrm{FP}\times \mathrm{FN}}{\sqrt{\left(\mathrm{TP}+\mathrm{FP}\right)\left(\mathrm{TP}+\mathrm{FN}\right)\left(\mathrm{TN}+\mathrm{FP}\right)\left(\mathrm{TN}+\mathrm{FN}\right)}}$	0.755
Cohen’s kappa	$\frac{2\left(\mathrm{TP}\times \mathrm{TN}-\mathrm{FP}\times \mathrm{FN}\right)}{\left(\mathrm{TP}+\mathrm{FP}\right)\left(\mathrm{FP}+\mathrm{TN}\right)+\left(\mathrm{TP}+\mathrm{FN}\right)\left(\mathrm{TN}+\mathrm{FN}\right)}$	0.754

## Data Availability

The data presented in this study are openly available via Zenodo (https://zenodo.org). The latest version of the curated dataset and the data enrichment attributes can be downloaded free of charge, using the following DOI: https://doi.org/10.5281/zenodo.10566883 The Enalos Asclepios KNIME nodes and workflow used in this study are proprietary to NovaMechanics Ltd. and require a licensing agreement for access.

## Supplementary Materials

The following supporting information can be downloaded at: https://www.mdpi.com/article/10.3390/ijms25105216/s1, Table S1: List of the 11 most significant molecular descriptors selected for model development; Table S2: List of the 41 compounds comprising the test set, the molecular descriptors’ values, and the obtained predictions using the proposed workflow; Dissemination of the predictive model using the QMRF reporting template (Table S3).

## Abbreviations

ACC	Accuracy
APD	Applicability domain
API	Application Programming Interfaces
CoMFA	Comparative molecular field analysis and
CoMSIA	Comparative molecular similarity indices analysis
DBD	DNA-binding domain
EC50	Half maximal effective concentration
FN	False negative
FP	False positive
GUI	Graphical user interface
HQSAR	Hologram quantitative structure–activity relationships
ISE	Iterative stochastic elimination
MCC	Matthews correlation coefficient
NIH	National Institutes of Health
NR	Nuclear receptor
OECD	Organisation for Economic Co-operation and Development
PFAS	Per- and polyfluoroalkyl substances
PPAR	Peroxisome proliferator-activated receptor
PPRE	Peroxisome proliferator response element
QMRF	QSAR model reporting format
QSAR	Quantitative structure–activity relationship
REST	Representational state transfer
RXR	Retinoid X receptor
S2k	Kier shape index (fixed length k = 2)
SDF	Structure-data file
SMILES	Simplified molecular input line entry system
TN	True negative
TP	True positive
kNN	k-nearest neighbours
κ	Cohen’s kappa
