# Supplementary material for: Development of a Robust Read-Across Model for the Prediction of Biological Potency of Novel Peroxisome Proliferator-Activated Receptor Delta Agonists
Source: Int J Mol Sci. 2024 May 10;25(10):5216. doi: 10.3390/ijms25105216 (PMC11121726; doi:10.3390/ijms25105216)
Supplement: Supplementary file 1 [file ijms-25-05216-s001.zip › ijms-2969211-supplementary.pdf]

Supplementary Information

Table S1: List of the 11 most significant molecular descriptors selected for model development.

Descriptor ID	Mold2 Descriptor	Description
D034	Number of Sulphur	Number of sulphur atoms in the molecule
D195	Maximal valence vertex electro-topological positive variation	The maximum positive value of ΔIi, where ΔIi >0 is the field effect on the ith atom due to the perturbation of the intrinsic state of the ith atom by all other atoms. dij refers to the topological distance between the ith and jth atom $\Delta I_i = \sum_{j=1} \frac{I_i - I_j}{\left(d_{ij} + 1\right)^2}$
D236	Kier 2-path index	Kier shape descriptor (κ), a topological shape index (derived from the Kier shape indices) defined in terms of the number of graph vertices and the number of paths mP with fixed length (m=2) in the H-depleted molecular graph
D280	Total information content order-5 index	The mth order (m=5) TICm represents a measure of the graph complexity, and it is defined as A (the number of graph vertices) times ICm (neighborhood Information Content) $TIC_m = A \cdot IC_m$
D301	Maximum eigenvalue weighted by Heteroatoms and Multiple bonds Matrix	Max eigenvalue - Modified (symmetric weighted) distance matrix accounting contemporarily for the presence of heteroatoms and multiple bonds in the molecule: $[ZD]_{ij}\{d_{ij}(Z,\pi^*), if\ i \neq j\ 1 - \frac{Z_C}{Z_i}, if\ i = j$ dij(Z, π*) is a weighted topological distance, Zc and Zi are the atomic numbers of the carbon and ith atoms respectively
D312	Sum eigenvalue weighted by electronegativity Pauling-Scale distance matrix	A spectral topochemical index that describes the alkyl groups. It is the sum of geometric means of the eigenvalues of the $^{a}A(\chi^{PA})$ augmented adjacency matrix weighted by the Pauling electronegativity scale (χ^{PA})
D375	Sum of topological distance between the vertices O and S	The sum of the distances (σi) of all the other atoms to a given atom, where dij is the topological distance between O and S atoms $\sigma_i \equiv \sum_{j=1}^{A} d_{ij}$
D377	Sum of topological distance between the vertices O and F	The sum of the distances (σi) of all the other atoms to a given atom, where dij is the topological distance between O and F atoms $\sigma_i \equiv \sum_{j=1}^{A} d_{ij}$
D437	Topological structure autocorrelation length-7 weighted by atomic Sanderson electronegativities (ATSe7)	Broto-Moreau spatial autocorrelation descriptors (ATS7) here weighted by atomic Sanderson electronegativities: $ATS_{7,e} = \frac{1}{2} \cdot \sum_{i=1} \sum_{j=1} w_i \cdot w_j \cdot \delta(d_{ij}; k)$
D573	Highest eigenvalue from Burden matrix weighted by van der Waals order 2	A Chemically Intuitive Molecular (CIM) index, the second largest positive eigenvalue of a modified connectivity matrix (Burden matrix B being a H-depleted molecular graph), determined by solving the general eigenvalue equation:
D746	Number of group H attached to C0(sp3) no X attached to next C	Number of Hydrogen groups attached next to a sp3 carbon atom where no other atoms were attached next to the Carbon atom

Table S2: List of the 41 compounds comprising the test set, the molecular descriptors' values and the obtained predictions using the proposed workflow.

CID	D034	D195	D236	D280	D301	D312	D375	D377	D437	D573	D746	Bind. Affin.*	Actual	Prediction
44626583	2	3.78	11.82	260.88	172.57	1.48	83	0	118.51	24.71	1	-9.0	Inactive	Inactive
44626585	2	3.81	11.15	241.79	154.67	1.48	77	0	113.84	24.73	1	-8.8	Inactive	Inactive
44626888	1	4.66	13.58	307.89	249.34	2.80	46	234	164.47	23.20	2	-9.5	Active	Active
44626689	1	3.75	11.56	251.83	184.81	1.73	46	0	135.24	23.20	1	-9.1	Active	Active
44626691	2	3.78	11.82	259.73	172.97	1.48	82	0	131.80	24.91	1	-8.8	Inactive	Inactive
44626789	0	3.73	11.30	249.08	191.03	1.97	0	0	136.96	23.15	1	-9.2	Active	Active
44626791	1	3.76	11.56	259.73	179.15	1.73	42	0	133.53	23.26	1	-9.0	Inactive	Active
46878727	2	3.69	14.80	293.36	243.36	1.93	119	0	138.58	24.71	0	-8.6	Inactive	Inactive
46878728	2	3.70	14.08	276.93	221.83	1.93	111	0	129.67	24.71	0	-8.3	Inactive	Inactive
46878729	2	3.71	13.37	258.70	201.23	1.93	103	0	118.11	24.71	0	-8.4	Inactive	Inactive
44626990	1	4.69	13.32	311.93	258.75	2.96	49	243	156.41	23.20	1	-10.4	Active	Active
46878777	2	3.77	12.02	242.94	170.79	1.67	83	0	109.52	24.71	0	-8.9	Inactive	Inactive
46878780	2	3.77	12.02	242.55	171.20	1.67	82	0	117.56	24.91	0	-8.7	Active	Inactive
46878843	1	3.75	11.75	242.55	177.38	1.92	42	0	119.27	23.28	0	-9.5	Inactive	Inactive
44627297	1	3.58	8.43	189.97	121.95	1.21	33	0	81.61	23.20	2	-9.1	Inactive	Inactive
44627192	1	4.52	11.13	251.60	194.77	2.87	39	195	127.82	23.15	1	-9.6	Active	Active
44627302	1	4.57	11.97	275.85	212.89	3.13	46	234	134.24	23.15	1	-9.9	Active	Active
44627409	1	4.67	8.93	197.97	150.29	2.29	33	183	85.89	23.20	1	-10.1	Inactive	Inactive
44627304	1	4.62	12.84	299.06	235.01	3.13	46	234	150.48	23.15	1	-9.8	Active	Active
44627306	1	4.68	13.24	316.51	259.36	3.29	49	243	153.46	23.15	1	-10.4	Active	Active
44627413	0	4.49	10.92	255.30	203.49	2.95	0	219	130.87	23.15	1	-9.9	Active	Active
44627300	1	3.61	8.64	195.90	123.02	1.21	33	0	92.47	23.18	2	-9.5	Inactive	Inactive
44627411	1	3.71	10.03	245.60	158.21	1.37	33	0	127.00	23.19	2	-9.2	Inactive	Inactive
44627415	0	4.55	11.76	281.65	221.92	3.21	0	258	137.44	23.15	1	-9.9	Active	Active
44627528	1	3.72	10.71	237.93	166.14	1.47	39	0	128.81	23.19	1	-9.1	Inactive	Inactive
44627628	1	3.72	10.26	219.89	155.88	1.40	33	0	123.70	23.36	1	-9.1	Active	Inactive
11678481	0	4.60	12.62	305.01	244.29	3.21	0	258	153.68	23.15	1	-9.9	Inactive	Active
44627417	0	4.65	13.03	323.28	267.69	3.37	0	267	156.59	23.15	1	-10.9	Active	Active
44627531	1	5.05	11.21	226.91	196.63	2.54	40	219	122.84	23.22	0	-9.7	Inactive	Inactive
44627729	1	5.08	11.43	257.25	203.34	2.54	40	219	138.96	23.22	1	-10.3	Active	Inactive
44627730	1	5.39	11.43	264.76	198.95	2.54	40	204	144.23	23.22	1	-9.4	Active	Inactive
44627839	1	4.59	12.05	261.83	213.67	2.80	46	234	137.19	23.20	1	-9.9	Active	Active
44627842	1	3.77	11.95	261.81	190.37	1.92	46	0	136.41	23.38	1	-9.1	Active	Active
44628050	0	3.72	10.85	242.89	180.23	1.91	0	0	131.53	23.19	1	-9.4	Active	Active
44626478	0	5.34	11.18	262.00	206.08	2.79	0	222	145.64	23.15	1	-10.0	Inactive	Active
44627731	1	5.09	11.45	271.05	208.14	2.29	32	174	139.68	23.22	2	-9.6	Active	Active
44627943	1	3.81	12.35	272.67	225.14	1.47	40	0	148.96	23.31	1	-10.4	Active	Active
44626480	0	3.75	12.00	284.87	205.54	1.71	0	0	148.30	23.15	4	-9.6	Active	Active
44626586	0	3.76	10.93	279.85	206.16	1.88	0	0	144.07	23.19	1	-10.6	Active	Active
44627847	1	5.08	11.00	238.01	190.56	2.48	30	168	115.64	23.38	0	-10.1	Inactive	Inactive
44626587	0	3.74	11.25	246.22	186.57	2.10	0	0	132.70	23.20	1	-9.6	Active	Active

* Binding affinities are calculated in kcal/mol

S1. Dissemination of the predictive model using the QMRF reporting template

**1.**	**QSAR identifier**	
1.1.	QSAR identifier (title)	Development of a robust read across model for the prediction of biological potency of novel PPARδ agonists with Isalos Analytics Platform
1.2	Other related models	Not Applicable
1.3.	Software coding the model	Isalos Analytics Platform (https://isalos.novamechanics.com/)<br>The model was implemented as a web application, available at: https://www.enaloscloud.novamechanics.com/scenarios/ppardelta/
**2.**	**General information**	
2.0	Abstract	A robust predictive model was developed using 136 novel peroxisome proliferator-activated receptor delta (PPARδ) agonists, a distinct subtype of lipid-activated transcription factors of the nu-clear receptor superfamily that regulate target genes by binding to characteristic sequences of DNA bases. The model employs various structural descriptors and docking calculations and provides predictions of the biological activity of PPARδ agonists, following the criteria established by (OECD).
2.1.	Date of QMRF	30 November 2023
2.2.	QMRF author(s) and contact details	Maria Antoniou – antoniou@novamechanics.com<br>Antreas Afantitis – afantitis@novamechanics.com
2.3.	Date of QMRF update(s)	Not Applicable
2.4.	QMRF update(s)	Not Applicable
2.5.	Model developer(s) and contact details	Maria Antoniou – antoniou@novamechanics.com<br>Konstantinos Papavasileiou – papavasileiou@novamechanics.com
2.6.	Date of model development and/or publication	Date of model development: July 2023.
2.7.	Reference(s) to main scientific papers and/or software package	Development of a Robust Read-Across Model for the Prediction of Biological Potency of Novel PPARδ Agonists, Int. J. Mol. Sci.
2.8.	Availability of information about the model	The model is non-proprietary: full description of the model algorithm is available; training and test sets are available as supplementary material of original research article. The model is implemented in a public web service and the full dataset with data enrichment attributes is publicly available at the following DOI: https://doi.org/10.5281/zenodo.10566883
2.9.	Availability of another QMRF for exactly the same model	Not Applicable
**3**	**Defining the endpoint - OECD Principle 1: "A DEFINED ENDPOINT"**	
3.1.	Species	Small Molecules
3.2.	Endpoint	Prediction of the potency class ('Active' or 'Inactive') of small molecules to peroxisome proliferator-activated receptor delta (PPARδ)
3.3	Comment on endpoint	Classified into two clusters based on the logarithmically transformed EC_{50} values of the initial compounds.
3.4.	Endpoint units	None
3.5.	Dependent variable	Two potency categories, 'Active' and 'Inactive'

3.6.	Experimental protocol	The dataset was sourced from the PubChem BioAssay repository, under the AID 469785. The bioassay evaluates the agonist activity at human PPARδ ligand binding domain and includes 136 novel oxazole-based compounds tested in human 293T cells co-transferred with Gal4-DBD via a process called luciferase transactivation. Each compound in the dataset is accompanied by measurements of the half maximal effective concentration (EC_{50}), a standardized type of potency.<br>More information on the experimental protocol can be found in: Epple, R.; Cow, C.; Xie, Y.; Azimioara, M.; Russo, R.; Wang, X.; Wityak, J.; Karanewsky, D.S.; Tuntland, T.; Nguyêñ-Trân, V.T.B.; et al. Novel Bisaryl Substituted Thiazoles and Oxazoles as Highly Potent and Selective Peroxisome Proliferator-Activated Receptor δ Agonists. J Med Chem 2010, 53, 77–105, https://doi.org/10.1021/jm9007399
3.7.	Endpoint data quality and variability	The log-transformed measurements of EC_{50} are partitioned into two clusters that represent two activity classes with a k-means clustering technique. Therefore, each compound is assigned a class label and the coverage of the two clusters, 'Active' and 'Inactive', is relatively balanced. The 'Inactive' category has a centroid of log(EC_{50})=0.224 and the 'Active' category has a centroid of log(EC_{50})=-1.674.
**4**	**Defining the algorithm - OECD Principle 2 : "AN UNAMBIGUOUS ALGORITHM"**	
4.1.	Type of model	Type of model: Non-parametric, supervised learning classification model
4.2.	Explicit algorithm	k-Nearest Neighbours algorithm (k = 8). This algorithm identifies the k-number of training data points that are 'closest' to the unclassified instance in the test set. Distances are assigned based on Euclidean distances, and inverted distance is used as weighting factor for the k nearest points.
4.3.	Descriptors in the model	Molecular descriptors refer to descriptors based on the one-dimensional and two-dimensional structure of a compound:<br>1. Number of sulfur atoms [D034]<br>2. Maximum valence vertex electro- topological positive variation [D195]<br>3. Kier 2-path index236 Kier 2-path index [D236]<br>4. Total information content order-5 index [D280]<br>5. Maximum eigenvalue weighted by heteroatoms and multiple bonds matrix [D301]<br>6. Sum eigenvalue weighted by electronegativity Pauling-Scale distance [D312]<br>7. Sum of topological distance between the vertices O and S [D375]<br>8. Sum of topological distance between the vertices O and F [D377]<br>9. Topological structure autocorrelation length-7 weighted by atomic Sanderson electronegativities (ATSe7) [D437]<br>10. Highest eigenvalue from Burden matrix weighted by Van der Waals order-2 [D573]<br>11. Number of group H attached to C0(sp3) no X attached next to C [D736]<br>12. Binding affinity calculations of the 136 compounds
4.4.	Descriptor selection	Feature selection was performed to select the most important variables from an initial pool of 777 molecular descriptors, using low-variance filters and column removal based on correlations between variables. The 'BestFirst (forward direction)' approach uses a greedy algorithm that iteratively adds or removes features to find the successor in all the tested combinations. 11 descriptors were selected, along with binding affinity calculations, a descriptor that was manually selected.
4.5.	Algorithm and descriptor generation	All of the 777 molecular descriptors were generated using the 'EnalosMold2' KNIME node that employs the Mold2.exe executable and an SDF file to calculate molecular descriptors.

4.6.	Software name and version for descriptor generation	Mold2 is a software developed by the National Center for Toxicological Research (NCTR) that calculates a large and diverse set of 777 molecular descriptors encoding two-dimensional chemical structure information. More information on the Mold2 software can be found in https://www.fda.gov/science-research/bioinformatics-tools/mold2
4.7.	Chemicals/Descriptors ratio	136 chemicals:12 descriptors
**5**	**Defining the applicability domain - OECD Principle 3: "A DEFINED DOMAIN OF APPLICABILITY"**	
5.1.	Description of the applicability domain of the model	The applicability domain is defined by fixed boundaries, the APD threshold, calculated by considering Euclidean distances between all molecules in the training set. If the domain of a molecule is beyond this threshold, then the prediction is considered unreliable.<br><br>More information on the applicability domain methodology can be found in: G. Melagraki, A. Afantitis, H. Sarimveis, P.A. Koutentis, O. Igglessi – Markopoulou, G. Kollias "In Silico Exploration for Identifying Structure–Activity Relationship of MEK Inhibition and Oral Bioavailability for Isothiazole Derivatives" Chemical Biology and Drug Design 2010; 76: 397–406, https://doi.org/10.1111/j.1747-0285.2010.01029.x
5.2.	Method used to assess the applicability domain	The distance of a test molecule to its nearest neighbour in the training set in compared to the pre-defined APD threshold, APD='d'+'stdev'*z.<br>• First, the average Euclidean distances between all pairs of training data is calculated and then the set of distances that were lower than the average is formulated.<br>• The 'd' and 'stdev' values are finally determined as the average and standard deviation of all distances included in the remaining set.<br>• z is an empirical parameter with a value of 0.5
5.3.	Software name and version for applicability domain assessment	'Domain-APD' node from the Enalos+ extension for the KNIME Analytics Platform (version 4.7.5)
5.4.	Limits of applicability	APD threshold=3.682, If the domain of a molecule is lower than this threshold, then the prediction is considered reliable. The reliability percentage of the testing set equals 100%.
**6**	**Defining goodness-of-fit and robustness (internal validation) – OECD Principle 4: "APPROPRIATE MEASURES OF GOODNESS-OF-FIT, ROBUSTENESS AND PREDICTIVITY"**	
6.1.	Availability of the training set	It is available but not attached. The training set can be derived from the supplementary material of the publication. The whole set of the filtered molecular descriptors can be accessed via Zenodo (https://doi.org/10.5281/zenodo.10566883).
6.2.	Available information for the training set	Available information for the training set:<br>a) SMILES notations<br>b) InChI and InChiKeys codes<br>c) PubChem Compound ID and ChEMBL ID.
6.3.	Data for each descriptor variable for the training set	The descriptor values of the training set are available via Zenodo.
6.4.	Data for the dependent variable for the training set	The dependent variable values of the training set are available via Zenodo.
6.5.	Other information about the training set	The Kennard-Stone algorithm was used for the partitioning of the initial dataset into two representative subsets, the training and the test set. The training set percentage was set at 70%, thus it consists of 95 small molecules selected for model development. Also, the training set includes 40 molecules characterised as 'Inactive', and 55 molecules characterised as 'Active'.

6.6.	Pre-processing of data before modelling	• Removal of columns: Filtering of columns that have the same values at a percentage equal or higher than 70%.<br>• Low Variance filter: Cutoff limit of 20% to filter out columns.<br>• Z-score normalization: Gaussian distribution.<br>• Separating the compounds into two clusters (k-means clustering)<br>• Feature selection was performed as described in Section 4.4.
6.7.	Statistics for goodness-of-fit	Confusion matrix, Accuracy, Sensitivity, Precision, F1-score, Cohen's kappa
6.8.	Robustness - Statistics obtained by leave-one-out cross-validation	Robustness statistics obtained by leave-one-out cross validation on the training set:<br>Accuracy=76.8%, Sensitivity=80%, Precision=80%, F1-score=80%, Cohen's kappa=0.525
6.9.	Robustness - Statistics obtained by leave-many-out cross-validation	Robustness statistics obtained by five-fold cross validation on the training set:<br>Accuracy=80%, Sensitivity=83.6%, Precision=82.1%, F1-score=82.9%, Cohen's kappa=0.588
6.10.	Robustness - Statistics obtained by Y-scrambling	Five Y-randomizations returned accuracies of 68.3%, 39.0%, 56.1%, 51.2%, 46.3%
6.11.	Robustness - Statistics obtained by bootstrap	Not Applicable
6.12.	Robustness - Statistics obtained by other methods	Not Applicable
**7**	**Defining predictivity (external validation) – OECD Principle 4: "APPROPRIATE MEASURES OF GOODNESS-OF-FIT, ROBUSTENESS AND PREDICTIVITY"**	
7.1.	Availability of the external validation set	It is available but not attached. The test set in available as a table as supplementary material of the publication. The table includes the data enhancement attributes of the test set, and more information can be accessed via Zenodo (https://doi.org/10.5281/zenodo.10566883).
7.2.	Available information for the external validation set	Available information for the training set:<br>a) SMILES notations<br>b) InChI and InChiKeys codes<br>c) PubChem Compound ID and ChEMBL ID.
7.3.	Data for each descriptor variable for the external validation set	The descriptor values of the test set are available via Zenodo.
7.4.	Data for the dependent variable for the external validation set	The dependent variable values of the test set are available via Zenodo.
7.5.	Other information about the external validation set	41 out of the 136 initial compounds were included in the test set. The test set was not involved in the model development, but it was used solely for validating purposes. Also, the test set includes 18 molecules characterised as 'Inactive', and 23 molecules characterised as 'Active'.
7.6.	Experimental design of test set	The initial dataset was split into a 70:30 ratio with the Kennard-Stone algorithm for the separation into two representative subsets.
7.7.	Predictivity - Statistics obtained by external validation	True Positives = 20, False Positives = 2, True Negatives = 16, False Negatives = 3, Accuracy = 87.8%, Sensitivity = 87.0%, Precision = 90.9%, F1-score = 88.9%, Matthew's correlation coefficient = 0.755, Cohen's kappa = 0.754
7.8.	Predictivity - Assessment of the external validation set	The external validation set is sufficiently large and representative of the original data. It was further assessed using the applicability domain limits. The external validation subset (test set) is 30% of the initial dataset, and all the instances of the test sets fall within the domain of applicability.
7.9.	Comments on the external validation of the model	Z-score normalization of the test set is applied based on the calculations on the training set.
**8**	**Providing a mechanistic interpretation - OECD Principle 5: "A MECHANISTIC INTERPRETATION, IF POSSIBLE"**	

8.1.	Mechanistic basis of the model	The 11 descriptors that better correlate the molecular characteristics of the compounds to the biological potency were selected among a pool of 777 variables and their influence on the end point was analysed. D280 (TCIm) is a variable that quantifies the complexity of a molecular graph, where higher values amount to higher effective concentrations. Also, the selected Broto-Moreau autocorrelation descriptor D437 (ATSe,7) is weighted by atomic electronegativities, and higher electronegativity distribution contributes to the activity of PPARδ. D236 is the Kier-shape index, which encloses information on the degree of star graph-likeness and the branching/flexibility of a molecule. It is a valuable descriptor in this study, since PPARδ activation is associated with flexible compounds and less pliable ones have lower potency.
8.2.	A priori or a posteriori mechanistic interpretation	A posteriori mechanistic interpretation: the selected descriptors' possible effect on the end point was analysed after the model development.
8.3.	Other information about the mechanistic interpretation	Not Applicable
**9**	**Miscellaneous information**	
9.1.	Comments	Not Applicable
9.2.	Bibliography	• Epple, R.; Cow, C.; Xie, Y.; Azimioara, M.; Russo, R.; Wang, X.; Wityak, J.; Karanewsky, D. S.; Tuntland, T.; Nguyễn-Trần, V. T. B.; Cuc Ngo, C.; Huang, D.; Saez, E.; Spalding, T.; Gerken, A.; Iskandar, M.; Seidel, H. M.; Tian, S.-S. Novel Bisaryl Substituted Thiazoles and Oxazoles as Highly Potent and Selective Peroxisome Proliferator-Activated Receptor δ Agonists. J Med Chem 2010, 53 (1), 77–105. https://doi.org/10.1021/jm9007399<br>• National Center for Biotechnology Information. PubChem Bioassay Record for AID 469785, Source: ChEMBL. https://pubchem.ncbi.nlm.nih.gov/bioassay/469785<br>• Mold(2), molecular descriptors from 2D structures for chemoinformatics and toxicoinformatics (Hong et al., 2008) DOI: 10.1021/ci800038f<br>• NovaMechanics Ltd, "Enalos + KNIME nodes," 2017. http://enalosplus.novamechanics.com/<br>• G. Melagraki, A. Afantitis, H. Sarimveis, P.A. Koutentis, O. Igglessi – Markopoulou, G. Kollias "In Silico Exploration for Identifying Structure–Activity Relationship of MEK Inhibition and Oral Bioavailability for Isothiazole Derivatives" Chemical Biology and Drug Design 2010; 76: 397–406, https://doi.org/10.1111/j.1747-0285.2010.01029.x<br>• Varsou, D.-D.; Tsoumanis, A.; Papadiamantis, A. G.; Melagraki, G.; Afantitis, A. Isalos Predictive Analytics Platform: Cheminformatics, Nanoinformatics, and Data Mining Applications; 2023; pp 223–242. https://doi.org/10.1007/978-3-031-20730-3_9.
9.3	Supporting information	Not Applicable
